# Advances in natural compounds modulating autophagy for the therapeutic intervention of heart failure

**DOI:** 10.1007/s11010-025-05473-y

**Published:** 2026-01-09

**Authors:** Jiaqian Tang, Chang Zhou, Mengyuan Li, Jing Tao, Ruying Deng, Xinyi Ouyang, Guomin Zhang, Huiping Liu

**Affiliations:** College of Integrative Medicine, Hunan University of Traditional Chinese Medicine, 300 Bachelor Street, Changsha, 410208 Hunan P.R. China

**Keywords:** Natural compounds, Heart failure, Autophagy, Signal pathway, Myocardial protection

## Abstract

Heart failure (HF) represents the terminal stage of multiple cardiovascular disorders and is characterized by substantial morbidity, disability, and mortality. Although standard pharmacotherapies offer partial symptomatic relief, their capacity to halt or reverse disease progression remains limited. In recent years, autophagy—an essential intracellular degradation and recycling process that safeguards cardiomyocyte homeostasis—has emerged as a critical determinant in the onset and progression of HF. In this review, we systematically examined approximately 170 research articles related to “natural compounds-autophagy-HF” published up to 2025, and synthesized major advances reported over the past five years. Based on chemical structure and natural source, representative bioactive compounds were categorized into five major classes: flavonoids, polyphenols, alkaloids, terpenoids, and marine-derived molecules. We then integrated evidence on the principal autophagy-regulatory signaling cascades influenced by these compounds, including the AMP-activated protein kinase (AMPK)/mechanistic target of rapamycin (mTOR) axis, phosphoinositide 3-kinase (PI3K)/protein kinase B (Akt), sirtuin 1 (SIRT1)/forkhead box O (FOXO), BCL2/adenovirus E1B 19 kDa-interacting protein 3 (BNIP3)/NIP3-like protein X (NIX), and Beclin-1 pathways. Moreover, this review summarizes how these natural compounds modulate autophagy to attenuate key pathological processes associated with HF, including cardiac hypertrophy, adverse ventricular remodeling, mitochondrial dysfunction, and cardiomyocyte apoptosis. By comparing convergent mechanisms and compound-specific differences in autophagy-mediated cardioprotection, we highlight emerging mechanistic patterns and therapeutic implications. Collectively, through structured classification of compounds, integration of autophagy signaling pathways, and assessment of translational potential, this review provides a systematic framework to guide the rational application and further development of natural products for the prevention and treatment of HF.

**Graphical abstract:**

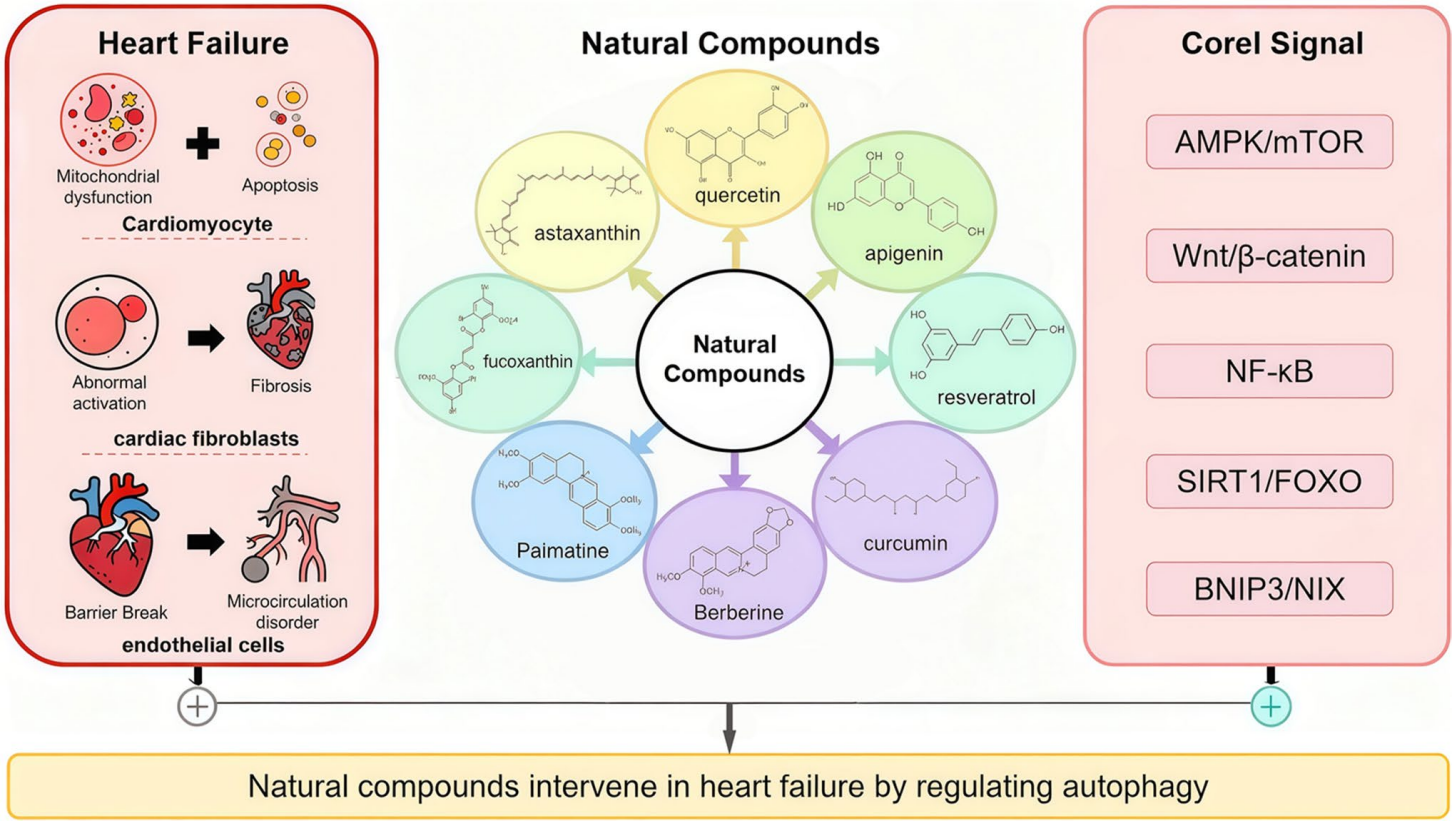

## Introduction

HF is a clinical syndrome caused by abnormal cardiac structure and/or function, resulting in decreased cardiac output and/or increased intracardiac pressure, which manifests as a series of symptoms and signs [1]. Despite substantial advances in pharmacological therapies and device-based interventions, the morbidity and mortality of HF remain high, particularly among the elderly population [2]. Therefore, the development of novel therapeutic strategies to improve patient prognosis is of great clinical importance.

Autophagy is a highly conserved intracellular degradation process that primarily utilizes the lysosomal pathway to remove damaged organelles and proteins, thereby maintaining cellular homeostasis [3]. Increasing evidence indicates that autophagy plays a critical role in HF by regulating the functional status of cardiomyocytes (CMs), cardiac fibroblasts (CFs), and cardiac endothelial cells (ECs). In CMs, moderate autophagy mitigates various cellular stresses, including ischemia, oxidative stress, and metabolic disturbances, thereby preserving cardiomyocyte function and delaying HF progression [4]. In contrast, excessive or insufficient autophagy can exacerbate myocardial injury, resulting in apoptosis or necrosis of CMs [5].

Autophagy also significantly influences CFs and ECs. In CFs, autophagy modulates activation and differentiation, regulating the secretion of collagen and other extracellular matrix (ECM) proteins, and thereby impacting cardiac fibrosis. For example, activation of the advanced glycation end-products (AGEs)-receptor for AGEs (RAGE) pathway induces autophagy in CFs, promotes differentiation into myofibroblasts, enhances pro-fibrotic activity, and exacerbates structural remodeling [6]. Conversely, inhibition of matrix metalloproteinase 9 (MMP9) enhances autophagic flux in CFs, restrains aberrant activation, and delays the progression of chronic HF [7]. In ECs, autophagy maintains vascular homeostasis by regulating oxidative stress, inflammatory responses, and nitric oxide (NO) production, while also suppressing endothelial-to-mesenchymal transition (EndMT), thereby limiting fibrotic transformation and cardiac fibrosis [8]. Collectively, autophagy orchestrates structural remodeling, metabolic homeostasis, and fibrotic processes across diverse cardiac cell types, positioning targeted modulation of autophagy as a promising therapeutic approach in HF.

Recently, the therapeutic potential of natural compounds in chronic disease management has garnered increasing attention, driven by multi-target intervention strategies and personalized treatment concepts. Natural products possess diverse chemical structures, broad biological sources, favorable biocompatibility, and low toxicity, making them suitable for long-term intervention in HF [9]. Numerous studies have demonstrated that natural compounds can modulate myocardial energy metabolism, oxidative stress, inflammation, and autophagy through multiple signaling pathways, thereby protecting CMs from injury [10]. Furthermore, the integration of pharmacology and systems biology has facilitated in-depth exploration of the mechanisms underlying natural product activity.

Compounds such as resveratrol, curcumin, and berberine have been shown to induce or restore moderate autophagy in CMs by activating AMP-activated protein kinase (AMPK) or sirtuin 1 (SIRT1), or by inhibiting mechanistic target of rapamycin (mTOR), ultimately improving ventricular remodeling and cardiac function [11]. Advances in structural optimization and novel drug delivery systems, including nanocarriers and liposomes [12, 13], have further enhanced pharmacokinetic profiles and tissue targeting, increasing their translational potential as therapeutic candidates. Taken together, natural products represent a promising area of research for HF intervention, both mechanistically and clinically.

Based on current research gaps and trends, this review systematically summarizes the progress of natural compounds in modulating HF through autophagy regulation. Unlike previous reviews that focus on single compounds or isolated pathways, this work categorizes natural products by structural class—including flavonoids, polyphenols, alkaloids, saponins, and marine-derived compounds—and synthesizes evidence from diverse HF models, including heart failure with reduced ejection fraction (HFrEF) and preserved ejection fraction (HFpEF). This approach highlights compound-specific characteristics, mechanistic insights, and translational potential for autophagy-mediated cardioprotection.

## Literature collection methods

The research data cited in this review were primarily retrieved from the bibliographic databases PubMed and Web of Science. Searches were conducted using combinations of the following keywords: specific natural compound names in conjunction with “autophagy,” “mitophagy,” or “autophagic flux,” and “HF,” “myocardial remodeling,” or related terms. The natural compounds considered include commonly studied bioactive ingredients such as flavonoids, polyphenols, alkaloids, and marine-derived compounds, as well as representative molecules including quercetin, apigenin, resveratrol, curcumin, berberine, palmatine, fucoxanthin, and astaxanthin.

The literature search was limited to publications from 2009 to 2025 to capture recent advances in the field of natural compounds–autophagy regulation–HF intervention. Nevertheless, to provide a comprehensive perspective on the historical development of the theoretical framework and underlying mechanisms, seminal studies of foundational significance outside this time window were also included when relevant. Representative studies were systematically collated and analyzed to summarize the autophagy-regulatory characteristics, target specificity, and translational potential of different classes of natural compounds in the context of HF. This approach allows for an integrated understanding of both mechanistic diversity and therapeutic applicability.

## Overview of autophagy

### Definition and classification

Autophagy is an evolutionarily conserved intracellular degradation process in eukaryotes, first proposed by Christian de Duve over 40 years ago [14]. It maintains cellular homeostasis by eliminating damaged organelles, misfolded proteins, and other intracellular wastes via lysosome-mediated pathways [15]. Based on the modes of cargo delivery to lysosomes, autophagy is generally classified into three types: macroautophagy, microautophagy, and chaperone-mediated autophagy (CMA) [16]. In macroautophagy, cytoplasmic components are sequestered within double-membrane vesicles termed autophagosomes, which subsequently fuse with lysosomes to form autolysosomes, where the contents are degraded and recycled for cellular reuse [17]. Microautophagy involves the direct invagination of the lysosomal membrane to engulf cytoplasmic material for degradation [18]. CMA is a highly selective process in which specific substrate proteins are recognized by chaperones and directly translocated into lysosomes for degradation [19]. Among these, macroautophagy is the most prevalent form and is the primary focus of this review [20].

### The process of autophagy

Autophagy proceeds through several sequential stages: induction, phagophore nucleation, phagophore elongation, autophagosome maturation, docking and fusion with lysosomes, and degradation followed by efflux of breakdown products [21]. It is activated in response to cellular stressors such as nutrient deprivation or energy deficit. AMP-activated protein kinase (AMPK) senses cellular energy status and initiates autophagy by directly phosphorylating the Unc-51 like autophagy activating kinase 1 (ULK1) complex [22]. Upon ULK1 activation, the class III phosphoinositide 3-kinase (PI3K-III) complex is recruited, and their interaction drives the nucleation of the phagophore [23]. During phagophore elongation, the autophagic membrane expands to form the isolation membrane. The ATG12–ATG5–ATG16L1 complex facilitates membrane elongation, while microtubule-associated protein 1 light chain 3 (LC3) is lipidated by phosphatidylethanolamine (PE) through the actions of ATG7 and ATG3 to form LC3-II, which participates in membrane expansion and closure [24]. Rab5 GTPases regulate the recruitment of the endosomal sorting complex required for transport (ESCRT) machinery to the phagophore, promoting its closure into a mature autophagosome [25]. Mature autophagosomes subsequently fuse with lysosomes to form autophagolysosomes [26]. Transcription factor EB (TFEB) plays a key role in substrate targeting and lysosomal degradation. Within autophagolysosomes, lysosomal hydrolases degrade autophagic cargo—including damaged organelles, proteins, and other macromolecules—into basic metabolites such as amino acids, fatty acids, and sugars, which are exported back to the cytoplasm for biosynthesis or energy metabolism, thereby maintaining cellular homeostasis [27].

## The pathological mechanism of HF and the role of autophagy

### Types of HF

HF is a complex and progressive clinical syndrome representing the final stage of various cardiovascular diseases. Its core pathological features include impaired ejection function, limited ventricular filling, and systemic hypoperfusion resulting from structural and/or functional cardiac abnormalities [28]. This reduced perfusion compromises the function of vital organs such as the brain, kidneys, and skeletal muscle, and may activate neurohormonal systems, including the renin–angiotensin–aldosterone system (RAAS) and the sympathetic nervous system (SNS), thereby promoting myocardial remodeling and further exacerbating HF progression [29]. The classification of HF is primarily based on left ventricular ejection fraction (LVEF). According to LVEF levels, HF is categorized into three major types: HFrEF [30], HFpEF [31], and HF with mildly reduced ejection fraction HFmrEF [32]. Each subtype exhibits distinct pathophysiological characteristics and clinical implications, which have important relevance for diagnosis, prognosis, and therapeutic strategies.

### Key cell autophagy in HF

#### Autophagy and mitochondrial quality control in cardiomyocytes

In CMs, autophagy serves as a critical quality control mechanism to maintain cellular homeostasis and energy balance. It preserves myocardial function and influences remodeling and survival by selectively removing damaged mitochondria, protein aggregates, and other dysfunctional organelles [33]. As a high-energy-demand tissue, mitochondria occupy approximately 30% of CM volume and provide ATP for contraction via oxidative phosphorylation [34]. Mitochondrial damage or dysfunction leads to excessive reactive oxygen species (ROS) production, lipid peroxidation, protein oxidative modifications, membrane dysfunction, and disrupted intracellular calcium homeostasis, resulting in impaired contractile function, metabolic imbalance, and potentially apoptosis or necrosis. To mitigate these damages, CMs activate mitophagy to remove impaired mitochondria and maintain metabolic homeostasis [35–37].

Mitophagy is primarily mediated via the ubiquitin-dependent PINK1/Parkin pathway and the ubiquitin-independent BNIP3/FUNDC1 pathway [38–40]. Under stress conditions such as hypoxia, pressure overload, or energy deprivation, these pathways synergistically identify and eliminate damaged mitochondria, thereby preserving myocardial energy metabolism and functional integrity [41]. However, sustained stress can disrupt this balance, leading to aberrant mitophagy and contributing to HF pathogenesis [42]. Animal models of pressure-overload-induced HF demonstrate that reduced PINK1/Parkin-mediated mitophagy is associated with CM apoptosis, mitochondrial dysfunction, and impaired cardiac performance [43]. Animal models of pressure-overload-induced HF demonstrate that reduced PINK1/Parkin-mediated mitophagy is associated with CM apoptosis, mitochondrial dysfunction, and impaired cardiac performance [42].

In summary, autophagy, particularly mitophagy, is central to CM energy homeostasis, oxidative damage limitation, and contractile preservation. Its dysregulation in HF exacerbates mitochondrial dysfunction, metabolic decline, cell death, and ventricular remodeling, highlighting restoration of moderate autophagy as a promising therapeutic strategy.

#### Autophagy regulation and anti-fibrosis in cardiac fibroblasts

CFs are the primary non-myocyte cell type in the cardiac interstitium, comprising 15–30% of adult cardiac cells [44]. Their principal functions include synthesis, degradation, and remodeling of the extracellular matrix (ECM) to maintain structural integrity and mechanical stability [45, 46]. Under homeostatic conditions, CFs coordinate collagen, elastin, and fibronectin production, and regulate the dynamic balance between matrix metalloproteinases (MMPs) and their endogenous inhibitors (TIMPs), maintaining ECM metabolic homeostasis and the myocardial microenvironment [47].

Upon stimulation by mechanical stretch, inflammatory mediators, or oxidative stress, resting CFs activate and differentiate into myofibroblasts, characterized by upregulation of *α*-smooth muscle actin (*α*-SMA) and type I/III collagen, resulting in excessive ECM deposition, reduced myocardial compliance, and diastolic dysfunction [48–52]. Persistent fibrosis disrupts mechanical and electrical coupling between CMs, promotes ventricular remodeling, and accelerates HF progression [49, 53, 54].

Autophagy is a key regulator of CF homeostasis and anti-fibrotic activity. It limits pro-fibrotic signaling, such as TGF-*β*1/Smad2/3, by removing damaged mitochondria and excessive ROS, thereby preventing CF differentiation into myofibroblasts [55, 56]. Additionally, autophagy degrades misfolded or unsecreted procollagen via lysosomes, maintaining ECM turnover and delaying remodeling and functional deterioration [57]. Endoplasmic reticulum (ER)-related autophagy selectively removes accumulated procollagen, mitigates ER stress, and prevents excessive ECM deposition [58, 59].

Overall, autophagy forms a central defense network against cardiac fibrosis through mitochondrial homeostasis, protein quality control, metabolic remodeling, and intercellular signaling, supporting myocardial structure and mechanical stability and providing a rationale for targeted anti-fibrotic interventions in HF.

#### Autophagy in endothelial cells and cardiac microcirculation stability

ECs constitute the structural core of cardiac microcirculation and serve as a critical barrier for vascular permeability, perfusion efficiency, and myocardial oxygen supply [60]. Autophagy is essential for EC homeostasis, structural integrity, and functional balance [61]. Impaired endothelial autophagy leads to accumulation of damaged mitochondria and ROS, reduced endothelial nitric oxide synthase (eNOS) activity, and insufficient NO production, resulting in microcirculatory dysfunction and compromised myocardial perfusion. Persistent hypoxia and metabolic stress within the myocardium are major drivers of ventricular remodeling and HF [62, 63]. In HFpEF patients, reduced coronary NO-dependent relaxation corroborates this mechanism, linking endothelial autophagy defects to impaired microvascular perfusion [64].

Autophagy preserves endothelial homeostasis through multiple processes. It regulates the dynamic turnover of cytoskeletal and adhesion proteins, such as VE-cadherin and PECAM-1, maintaining vascular barrier stability [65]. EC-specific Atg5 knockout models exhibit connexin aggregation and increased capillary permeability, leading to impaired myocardial perfusion, tissue hypoxia, metabolic stress, and accelerated HF progression [61, 66]. Moreover, autophagy mitigates ROS accumulation and preserves eNOS-NO axis functionality via mitochondrial quality control, ensuring microvascular diastolic capacity. Impaired endothelial autophagy not only exacerbates microcirculation defects but also amplifies myocardial hypoxia and functional impairment, thereby accelerating HF progression [62].

**Fig. 1:**
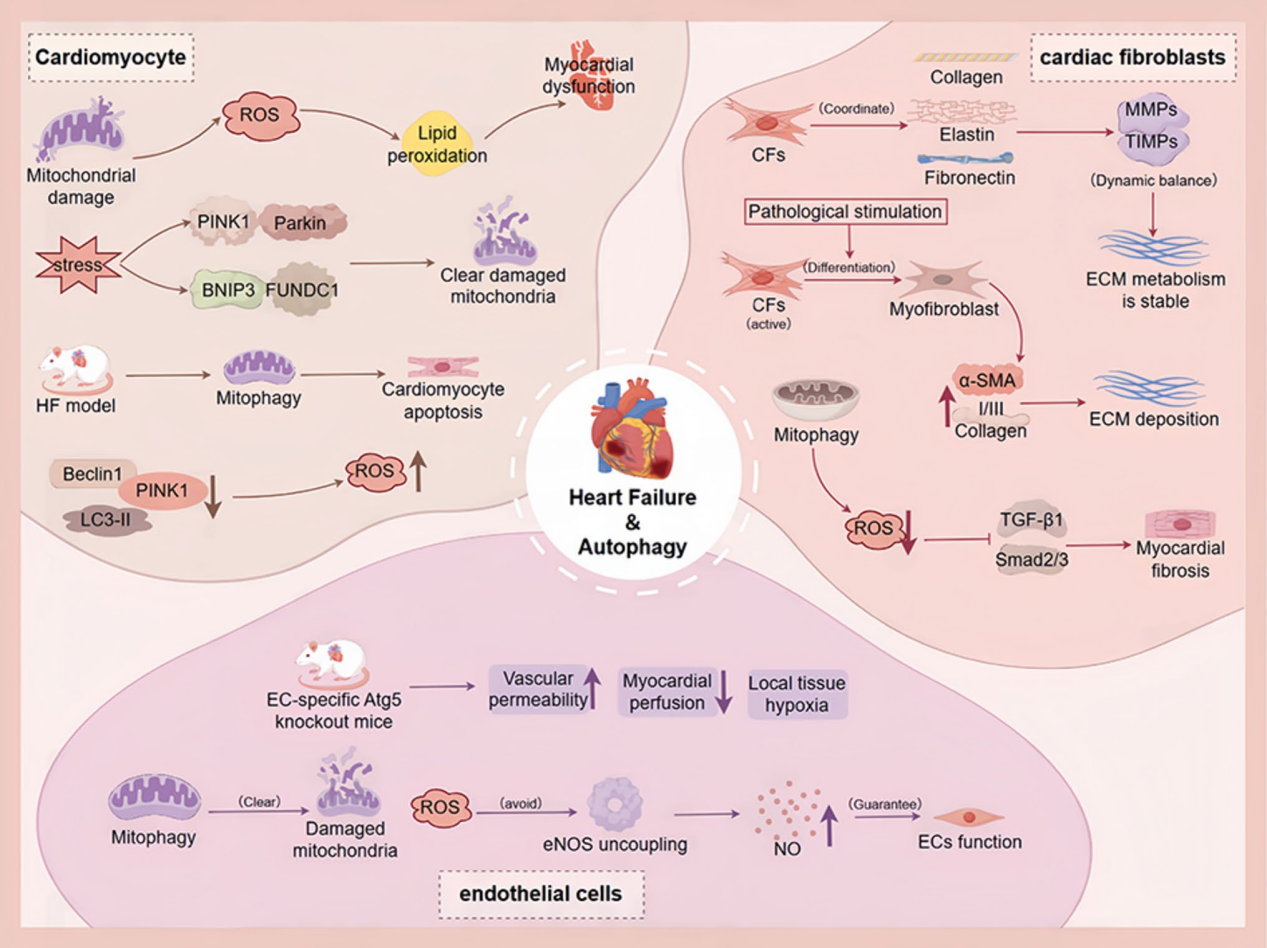
Overview of autophagy-related mechanisms contributing to heart failure. The diagram summarizes how impaired mitophagy, mitochondrial dysfunction, and excessive ROS production affect cardiomyocytes, cardiac fibroblasts, and endothelial cells. Disrupted mitochondrial quality control promotes cardiomyocyte. apoptosis and contractile dysfunction, enhances fibroblast activation and extracellular matrix deposition, and impairs endothelial homeostasis, collectively driving heart failure progression

In summary, endothelial autophagy is a core regulator of cardiac microcirculation homeostasis. Its dysfunction contributes to insufficient myocardial perfusion and HF pathogenesis, highlighting the therapeutic potential of strategies aimed at restoring or enhancing endothelial autophagy. As shown in Fig. 1.

### Autophagy-related pathways

#### AMPK–mTOR signaling in autophagy regulation during HF

The AMPK–mTOR signaling axis is central to autophagy regulation and myocardial remodeling in CFs. AMP-activated protein kinase (AMPK) functions as an energy sensor, detecting changes in the AMP/ATP ratio. The AMPK complex comprises a catalytic *α* subunit and regulatory *β* and *γ* subunits. Under energy depletion or stress, the *α* subunit is phosphorylated at Thr172, while the *γ* subunit binds AMP or ADP to stabilize the active conformation [67, 68]. Activated AMPK inhibits mTORC1 activity by phosphorylating TSC2 and Raptor, thereby relieving mTORC1-mediated suppression of autophagy [69]. mTORC1 is a classical negative regulator of autophagy, and its inhibition significantly enhances autophagic activity. AMPK activators, such as AICAR and A769662, suppress CF proliferation and type I/III collagen expression, reducing cardiac fibrosis [70]. Mechanistically, AMPK activation promotes autophagosome formation and autophagic flux by inhibiting phosphorylation of downstream mTORC1 targets 4EBP1 and p70S6K, accompanied by upregulation of LC3-II and Beclin-1 and downregulation of p62, indicating restored or enhanced autophagic flux [71, 72].

In HF, myocardial energy metabolism remodeling and mitochondrial dysfunction are core pathological events. The AMPK/mTORC1-regulated autophagy pathway mitigates ventricular remodeling and HF progression by maintaining energy homeostasis, removing damaged organelles, and reducing CF activation [73, 74]. Therefore, enhancing CF autophagy via AMPK activation or mTORC1 inhibition represents a potential therapeutic strategy for HF.

#### Bidirectional regulation of the Wnt/*β*-catenin pathway in HF

Wnt/*β*-catenin signaling is closely linked to autophagy and CF activation. Under energy stress or nutrient deprivation, *β*-catenin can bind LC3, forming a complex targeted for autophagic degradation, thereby inhibiting its transcriptional activity [75, 76]. Reciprocal regulation exists: Wnt activation suppresses p62 expression, while enhanced autophagy promotes *β*-catenin degradation, forming a negative feedback network. In HF, Wnt/*β*-catenin signaling drives ECM accumulation by upregulating IL-11 and enhancing TGF-*β*-mediated CF activation, accelerating remodeling and HF progression [77]. CF-specific *β*-catenin deficiency reduces pressure overload-induced fibrosis and preserves cardiac function [78].

Thus, autophagy-mediated *β*-catenin degradation limits CF activation and ECM accumulation, potentially slowing HF-related remodeling and providing a mechanistic basis for therapeutic targeting of the AMPK/mTOR and Wnt/*β*-catenin axes.

#### Autophagy–NF-κB crosstalk in cardiac inflammation and HF progression

NF-κB signaling is pivotal in cardiac inflammation, apoptosis, and structural remodeling [79]. Both clinical and experimental data indicate that the severity of cardiac inflammation correlates with HF progression, highlighting the key role of immune responses [80]. In pressure-overloaded hearts, NF-κB activation in CFs recruits inflammatory Ly6C^hi monocytes, exacerbating myocardial inflammation and dysfunction; conversely, NF-κB inhibition attenuates inflammatory infiltration and improves ventricular function [81].

Autophagy modulates NF-κB activity in a protective manner. Oxidative stress induced by H_2_O_2_ triggers autophagy-dependent apoptosis of cardiac stem cells via the ROS/NF-κB/NR4A2 pathway, while autophagy inhibition attenuates NF-κB activation and reduces apoptosis [82]. Mitophagy limits excessive NF-κB signaling by removing damaged mitochondria and inflammatory mediators, mitigating inflammation and fibrosis [83].

Therefore, autophagy restrains cardiac inflammation and fibrosis by negative regulation of NF-κB, preserves CM survival and mitochondrial function, and stabilizes ventricular structure. Enhancing autophagy or specifically modulating NF-κB may serve as a viable therapeutic approach to delay HF progression.

#### SIRT1/FOXO-mediated epigenetic regulation of autophagy in HF

SIRT1, a NAD^+^-dependent deacetylase, regulates metabolism, stress response, and autophagy. It deacetylates FOXO transcription factors (e.g., FOXO1, FOXO3), enhancing their binding to autophagy-related genes such as LC3 and BNIP3, thereby upregulating transcriptional activity. SIRT1 also deacetylates key ATG proteins (ATG5, ATG7, ATG8), promoting autophagosome formation [84]. In HF, CMs are exposed to ischemia, oxidative stress, and metabolic disorders, leading to mitochondrial damage and protein aggregation. Moderate autophagy, driven by SIRT1/FOXO, maintains CM homeostasis by clearing damaged mitochondria and abnormal proteins, reducing injury, and delaying ventricular remodeling and HF progression [85]. This pathway represents a mechanistic basis for potential autophagy-targeted interventions in HF.

#### BNIP3/NIX axis-mediated hypoxic mitophagy in HF

BNIP3 and NIX (BNIP3L) are mitochondrial outer membrane receptors that recruit LC3 via their LC3-interacting region (LIR), mediating selective mitophagy of damaged mitochondria. Under hypoxia, mechanical stress, or metabolic stress, BNIP3/NIX expression is upregulated, promoting clearance of dysfunctional mitochondria, limiting ROS accumulation, and preventing CM apoptosis [86].

**Fig. 2:**
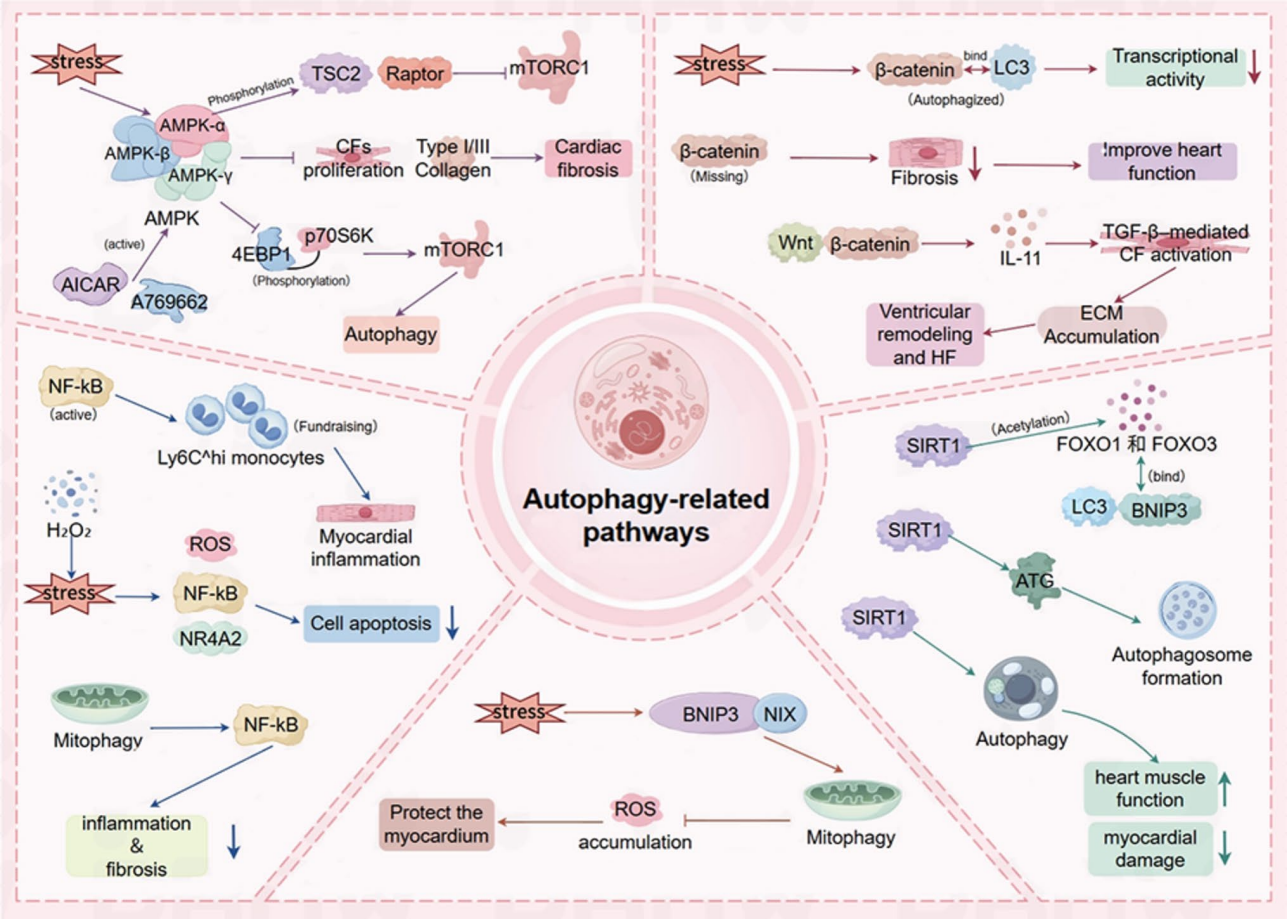
Overview of autophagy-related signaling pathways involved in cardiac remodeling andheart failure. The schematic highlights key regulatory nodes modulating autophagy across cardiomyocytes, immune cells, fibroblasts, and myocardial tissue, including stress-responsive pathways, metabolicsignaling, inflammatory regulation, and mitophagy-associated factors

Given the high sensitivity of CMs to mitochondrial dysfunction, BNIP3/NIX-mediated mitophagy is critical for maintaining energy homeostasis and redox balance. In models of myocardial ischemia–reperfusion and pressure overload-induced HF, activation of this pathway confers cardioprotection by removing damaged mitochondria and preserving metabolic function [87]. Thus, enhancing BNIP3/NIX-dependent mitophagy represents a potential therapeutic target for mitigating HF progression under hypoxic and metabolic stress conditions. As shown in Fig. 2.

## Research progress of multiple natural compounds regulating HF through autophagy

### Flavonoids

#### Quercetin

Quercetin is a polyhydroxyflavonoid widely distributed in natural foods, including onions, apples, green tea, grapes, and berries [88]. Its molecular formula is C_15_H_10_O_7_, comprising the typical A, B, and C ring structures. The 3′,4′-dihydroxyl groups of the B ring and the 3-hydroxyl/4-ketone conjugate system of the C ring constitute the key pharmacophore responsible for antioxidant activity and target protein interactions [89]. Molecular docking analyses indicate that quercetin can form hydrogen bonds and hydrophobic interactions with the CBS (Bateman) domain of the AMPK *γ* subunit, promoting AMPK phosphorylation and activation [90]. This activation inhibits downstream mTORC1, enhances ULK1 complex activity, and thereby increases autophagic flux, contributing to improved myocardial energy homeostasis. In a type II diabetic rat myocardial injury model, quercetin treatment significantly improved myocardial structure and function, upregulated LC3 and Beclin-1 expression, and reduced p62 accumulation, suggesting enhanced autophagy via the AMPK/mTORC1 pathway [72]. In addition, SIRT1 promotes PGC-1*α* deacetylation and activation. Activated PGC-1*α* enhances mitochondrial biogenesis and oxidative phosphorylation, improves energy supply, and positively regulates AMPK phosphorylation while inhibiting mTORC1 activity, thereby synergistically promoting autophagy and mitophagy [91–93].

In summary, quercetin exerts multi-level regulation by establishing an upstream molecular regulatory environment through antioxidant and anti-inflammatory effects and directly activating the SIRT1/PGC-1*α*/AMPK/mTORC1-mediated autophagy network. This mechanism ensures mitochondrial quality control, restores energy homeostasis, and systematically protects CMs in HF.

#### Apigenin

Apigenin is a natural flavonoid widely distributed in celery, chamomile, onion, and citrus fruits [94], exhibiting anti-inflammatory, anti-fibrotic, and cardioprotective effects [95]. Its flavonoid skeleton contains multiple hydroxyl substituents at the 4′, 5, and 7 positions, increasing polarity and hydrogen bond-forming potential, thereby enhancing binding to membrane proteins and protein–protein interaction interfaces [96]. Molecular docking and kinetic analyses indicate that apigenin can form stable complexes with several cardiovascular-related kinases: binding energies with p38 MAPK and GSK-3*β* are approximately − 8.21 and − 8.28 kcal·mol^−1^, respectively, with hydroxyl groups forming hydrogen bonds with residues such as Ile62, Glu97, and Val135, while the aromatic rings contribute to hydrophobic/π–π stacking [97, 98]. For AKT1, binding energy is around − 7.36 kcal·mol^−1^, with hydrogen bonds forming with Ala58, Asn53, and Asn199 in the ATP-binding pocket, potentially competing with ATP binding [99]. These results suggest high binding stability and significant biological activity at the interface of membrane proteins and signaling kinases.

Apigenin mediates cardioprotection through the regulation of autophagy and mitochondrial homeostasis via multiple signaling pathways. It activates the AMPK/mTOR axis and modulates SIRT1 activity, thereby maintaining autophagic flux [100]. Enhanced autophagy is evidenced by increased LC3-II/LC3-I ratio and accelerated p62 degradation, ensuring efficient autophagosome–lysosome fusion and the clearance of damaged mitochondria and cellular substrates [26, 101]. Mitophagy improves mitochondrial membrane potential and ATP production, contributing to myocardial energy homeostasis [102]. Furthermore, apigenin promotes the activity of TFEB, a key transcription factor in the autophagy–lysosomal pathway, stimulating lysosomal biogenesis, autophagosome formation, and autophagosome–lysosome fusion, thereby facilitating substrate clearance [103]. In LPS-induced cardiotoxicity models, apigenin enhances TFEB nuclear translocation, upregulates downstream targets Vps11 and Map1lc3, and activates the TFEB-mediated autophagy–lysosomal pathway to preserve myocardial structure and function [104]. Although clinical trials are lacking, current preclinical studies suggest broad potential for cardiovascular protection.

In summary, apigenin exerts myocardial protective effects through multi-level regulation of signaling pathways, autophagy–lysosome activity, and mitochondrial function, providing a mechanistic basis for potential heart failure interventions.

### Polyphenols

#### Resveratrol

Resveratrol is a natural polyphenolic compound abundant in grapes, peanuts, and red wine, exhibiting potent cardioprotective activities [105]. Structurally, its two benzene rings linked by a vinyl bridge, together with the conjugated 3,5,4′-hydroxyl groups, confer strong free radical–scavenging properties and high affinity for protein active sites [106, 107]. Structure–activity analyses further indicate that the 4′-hydroxyl group is essential for SIRT1 binding, interacting with Asp292 in its catalytic pocket to stabilize the enzyme conformation and enhance deacetylase activity [108, 109]. Activated SIRT1 promotes autophagy by deacetylating Atg5/Atg7 [110, 111] and transcription factors FoxO1/FoxO3a, thereby upregulating autophagy-related genes and stimulating mitophagy [112, 113]. Consistently, in neonatal rat cardiomyocytes subjected to hypoxia/reoxygenation, resveratrol activates the SIRT1/SIRT3–FoxO axis, increases LC3-II levels, and facilitates p62–Parkin-mediated removal of dysfunctional mitochondria [114]. Additional in vitro evidence demonstrates enhanced LC3-II/LC3-I conversion, autophagosome maturation, and BNIP3L-dependent mitophagy [115], supporting the establishment of a complete SIRT1/FoxO-mediated mitophagy regulatory cascade.

Beyond SIRT1, resveratrol exerts cardioprotection through multiple complementary signaling pathways. Under energy stress, it promotes phosphorylation of AMPK at Thr172, thereby maintaining autophagic flux and mitochondrial quality control, a mechanism functionally similar to that of quercetin [116]. Additionally, resveratrol activates the Nrf2-dependent antioxidant program [117]. The Nrf2–p62 positive feedback loop has been proposed to enhance cellular antioxidant capacity by promoting Keap1 degradation and activating autophagy-related gene transcription [118]; however, whether resveratrol fully engages this loop in cardiomyocytes requires further validation.

Collectively, resveratrol functions as a prototypical multi-target autophagy modulator capable of orchestrating SIRT1, AMPK, and Nrf2 signaling to support mitochondrial homeostasis and metabolic remodeling in the failing myocardium. Despite robust preclinical evidence demonstrating its autophagy-dependent cardioprotective efficacy, clinical translation remains limited by poor oral bioavailability, rapid systemic metabolism, and insufficient target specificity. Future work should prioritize pharmacokinetic optimization, rational structural modification, and combinatorial therapy strategies to facilitate its progression from experimental research toward clinical application.

#### Curcumin

Curcumin, a natural polyphenolic compound derived from *Curcuma longa*, possesses prominent antioxidant [119], anti-inflammatory [120], and anti-tumor [121] properties. Its molecular structure comprises two ortho-methoxy phenolic hydroxyl groups and an *α*,*β*-unsaturated *β*-diketone linker, which endow it with strong free radical–scavenging and metal-chelating capacity, while also providing structural compatibility with diverse protein active pockets [122]. Structure–activity investigations show that the phenolic hydroxyl groups of curcumin can form a covalent bond with the critical Cys151 residue of Keap1 via Michael addition, thereby destabilizing the Keap1-Nrf2 complex, promoting Nrf2 cytoplasmic release and nuclear translocation, and initiating transcriptional activation of antioxidant and autophagy-related genes such as *HO-1* and *NQO1* [123, 124]. Meanwhile, the *β*-diketone moiety enhances cellular energetic status and increases LC3 expression through metal ion chelation and ROS attenuation, contributing to improved autophagic flux [125, 126]. In a high-fat diet–induced diabetic cardiomyopathy model, curcumin inhibits mTORC1 activation, increases the LC3-II/I ratio, reduces p62 accumulation, and promotes clearance of dysfunctional mitochondria and protein aggregates, effectively alleviating cardiomyocyte apoptosis and ventricular remodeling [127].

In addition to Nrf2 activation, curcumin modulates several other autophagy-associated pathways relevant to cardiac protection. In myocardial aging models, curcumin activates the SIRT1/AMPK axis while suppressing mTOR phosphorylation, thereby enhancing autophagy and reducing aging-related markers including p53, p16, and ROS [128]. Conversely, in doxorubicin (DOX)-induced cardiotoxicity, curcumin activates the PI3K/Akt/mTOR pathway to restrain excessive autophagy-associated injury, highlighting its bidirectional regulatory nature—promoting adaptive autophagy to preserve homeostasis while preventing deleterious overactivation [129].

Overall, curcumin represents a quintessential multi-target autophagy modulator capable of synchronizing oxidative stress reduction, energy metabolism improvement, and signaling network stabilization, thereby providing comprehensive cardioprotection in the pathological milieu of heart failure. Nevertheless, the clinical potential of curcumin remains constrained by poor bioavailability and metabolic instability. Future research integrating nano-delivery platforms, rational structural modification, and combination strategies targeting autophagy could further enhance its specificity, efficacy, and therapeutic translational value.

### Alkaloids

#### Berberine

Berberine (BBR) is a representative isoquinoline alkaloid mainly derived from medicinal plants such as *Coptis chinensis*, *Phellodendron amurense*, and *Berberis vulgaris* [130]. Its chemical structure consists of a planar tetracyclic isoquinoline core containing a quaternary ammonium cation, forming a stable conjugated system that enables strong interactions with biomembranes and protein active sites [131]. Structure–activity relationship (SAR) analyses indicate that the positively charged quaternary ammonium nitrogen and substitutions at adjacent positions (e.g., C-8/C-9) play essential roles in biological activity. Multiple SAR studies demonstrate that retaining the quaternary ammonium moiety while introducing methoxy or benzyloxy groups at ortho positions significantly enhances target affinity and cellular activity [132, 133]. Molecular docking and virtual screening further reveal that berberine and its derivatives can form stable complexes with various signaling proteins, and that modifications in charge distribution or substitution patterns alter binding energy and complex stability [134–136]. Collectively, the quaternary ammonium–based positive charge and ortho substitutions confer extensive signaling regulatory potential by reinforcing electrostatic, hydrogen-bonding, and hydrophobic interactions—providing a molecular foundation for berberine’s multi-target roles in HF intervention.

Functionally, berberine improves myocardial performance by modulating energy-sensing and autophagy-related signaling pathways [137]. In the transverse aortic constriction (TAC) model, berberine suppresses mTORC1 phosphorylation and downstream effectors p70S6K and 4E-BP1, thereby attenuating myocardial hypertrophy and functional impairment [138]. Berberine also accumulates in mitochondria and elevates the NAD^+^/NADH ratio, leading to activation of the SIRT1 pathway [139–142]. In HFpEF models, berberine markedly upregulates PGC-1*α* expression, restores mitochondrial homeostasis, and reduces cardiomyocyte apoptosis [143]. Concurrently, berberine activates Parkin-dependent mitophagy, promoting the clearance of damaged mitochondria, improving metabolic derangements, and mitigating myocardial remodeling and dysfunction in TAC-induced HF [144].

In summary, berberine exerts significant cardioprotective effects during HF progression by coordinating mitochondrial quality control and energy metabolism through multi-target regulation of AMPK/mTOR, SIRT1/PGC-1*α*, and Parkin signaling axes. Its multi-pathway modulation combined with its modifiable structural features underscores its therapeutic promise in HF prevention and treatment. However, low oral bioavailability and rapid systemic metabolism still hinder clinical translation. Future research should focus on structural optimization, advanced delivery systems, and combination therapy strategies to facilitate the transition of berberine from experimental studies to clinical application.

#### Palmatine

Palmatine (PLT) is a typical isoquinoline alkaloid widely distributed in *Coptis chinensis* and other traditional medicinal plants, exhibiting notable anti-inflammatory, antioxidant, and cardioprotective properties [145]. From a structure–activity relationship perspective, its isoquinoline nucleus and methoxy substituents are regarded as key determinants of bioactivity [146, 147]. These structural features enhance hydrophobicity and conjugation stability, enabling specific interactions with biomembranes and diverse protein active sites. Further studies indicate that palmatine can intercalate into the DNA double helix and bind key signaling proteins, thereby regulating NF-κB/NLRP3, Nrf2/HO-1, and AMPK/mTOR pathways through multi-target mechanisms [148]. In HF-related experimental models, palmatine ameliorates cardiac dysfunction, reduces inflammation and oxidative stress, and induces protective autophagy via SIRT1 activation in doxorubicin (DOX)-induced myocardial injury, thereby exerting significant cardioprotective effects [145, 149].

Overall, palmatine, as a representative isoquinoline alkaloid, contributes to the maintenance of myocardial energetic and structural homeostasis by integrating SIRT1, AMPK/mTOR, Nrf2, and other signaling pathways through synergistic anti-inflammatory, antioxidant, and autophagy-regulating effects. These mechanistic insights provide a theoretical basis for its potential application in HF therapy. Nevertheless, current research remains primarily limited to in vitro and animal studies, and its pharmacokinetic characteristics, tissue targeting, and long-term safety require further investigation. Future efforts should emphasize structural optimization, development of advanced drug delivery systems, and exploration of combination therapeutic strategies to support its clinical translation in cardiovascular disease management.

### Novel marine-derived compounds

#### Fucoxanthin

Fucoxanthin (FX) is a non–vitamin A marine carotenoid widely distributed in the chloroplasts of algae and diatoms, belonging to lutein-derived xanthophylls [150, 151]. It exhibits multiple biological functions, including antioxidant [152], anti-inflammatory [153], and metabolic regulatory activities [154]. Structurally, FX contains an allenic bond, an allylic acetylene moiety, a 5,6-epoxy group, multiple conjugated double bonds, and oxygen-containing functional groups [155]. These unique features confer strong free radical–scavenging capacity [156] and promote mitophagy while ameliorating mitochondrial dysfunction across various models [157, 158]. Although current studies support the contribution of its overall molecular architecture to biological activity, systematic evidence regarding site-specificity and structure–activity relationships (SAR) in myocardial autophagy and mitophagy remains limited, underscoring the need for targeted chemical modification combined with in vivo HF models.

In HF-related experimental systems, FX demonstrates pronounced cardioprotective effects. In rat myocardial ischemia/reperfusion (MI/R) and streptozotocin-induced diabetic cardiomyopathy (STZ-DCM) models, FX activates AMPK and subsequently modulates GSK-3*β*/AKT and Nrf2/HO-1 signaling axes, thereby reducing oxidative stress, suppressing inflammation and apoptosis, and alleviating myocardial injury and hypertrophy [159, 160]. Moreover, FX upregulates the BNIP3/NIX pathway to enhance the clearance and functional restoration of damaged mitochondria, ultimately improving cardiomyocyte energy metabolism [161].

Overall, FX exerts antioxidant, anti-inflammatory, antifibrotic, and cardioprotective effects in multiple HF models through regulation of oxidative homeostasis and autophagy/mitophagy, attributed to its distinct molecular characteristics. However, the structure-dependent mechanisms underlying autophagy and mitophagy remain incompletely defined. Future studies incorporating rational chemical modification and in vivo HF models are needed to elucidate key structural sites and molecular mechanisms, thereby providing a theoretical basis for the clinical translation of FX in HF prevention and therapy.

#### Astaxanthin

Astaxanthin (AST) is a natural carotenoid primarily derived from the microalga *Haematococcus pluvialis*, the yeast *Xanthophyllomyces dendrorhous*, and marine crustaceans, and various marine crustaceans [162]. Its molecular structure features long-chain conjugated double bonds and terminal hydroxyl/ketone groups, which not only confer potent antioxidant properties but also facilitate its insertion into cardiomyocyte mitochondrial membranes, enabling regulation of membrane-associated signaling pathways [163]. These structural attributes underpin the myocardial protective and autophagy-modulating actions of AST. Experimental studies indicate that AST can initiate or restore autophagic flux through AMPK activation [164, 165].

Evidence from myocardial injury models supports the autophagy-regulating role of AST. In DOX-induced cardiotoxicity models, AST pretreatment increases p62 levels while decreasing Beclin-1 and LC3 expression [166]. Similarly, in H9c2 cardiomyocytes, AST mitigates DOX-induced cytotoxicity, reduces ROS accumulation, and restores mitochondrial function [167].

**Fig. 3:**
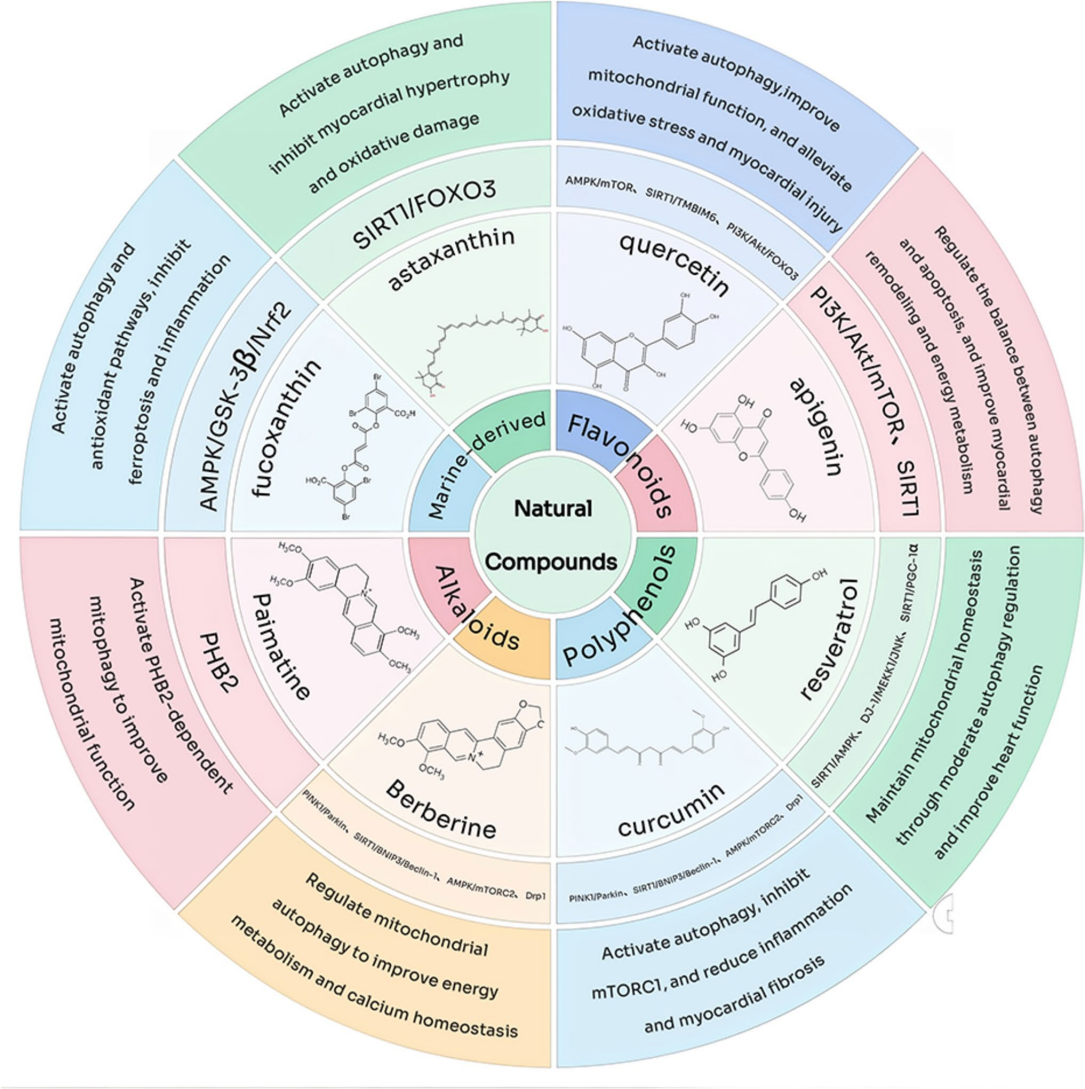
Overview framework of natural compounds

Despite these promising findings, several limitations persist. Most studies rely on acute injury or drug-toxicity models rather than chronic left ventricular remodeling or pressure overload-induced HF models. Additionally, many investigations do not comprehensively assess autophagic flux or mitophagy, nor do they integrate autophagy with assessments of energy metabolism, calcium homeostasis, or apoptosis. Thus, although AST exhibits potential in the context of HF, its mechanisms require further systematic validation. Future work should prioritize chronic HF models, dynamic evaluation of autophagic flux, detailed characterization of mitophagy, and combination strategies with established HF therapies to comprehensively determine its translational value. As shown in Fig. 3, Tables 1 and 2.

**Table 1:** Natural compounds regulating autophagy in heart failure

Compound	Source	Signaling pathways & mechanisms	HF model	Main outcomes	References
Quercetin	Onion, apple, green tea, grape, berries	AMPK/mTORC1: Activates AMPK *γ* subunit → inhibits mTORC1 → activates ULK1 complex → enhances autophagic flux SIRT1/PGC-1*α*/AMPK:SIRT1 deacetylates PGC-1*α* → enhances mitochondrial biogenesis & oxidative phosphorylation → positively regulates AMPK → inhibits mTORC1 → promotes mitophagy	Type 2 diabetes rat model	↑LC3, ↑Beclin-1; ↓p62; improved mitochondrial function and myocardial energy homeostasis; protected cardiac structure and function	[88–93]
Apigenin	Celery, chamomile, onion, citrus fruits	AMPK/mTOR: Activates AMPK → inhibits mTOR → promotes autophagic flux SIRT1: Activates SIRT1 → deacetylates autophagy-related proteins TFEB-autophagy–lysosome pathway: Promotes TFEB nuclear translocation → enhances autophagosome formation and autophagosome–lysosome fusion → facilitates clearance of damaged mitochondria	LPS-induced cardiotoxicity model	↑LC3-II/I; ↓p62; improved mitochondrial clearance and ATP production; preserved cardiac structure and function	[26, 94–104]
Resveratrol	Grape, peanut, red wine	SIRT1/FoxO:SIRT1 deacetylates FoxO1/FoxO3a → upregulates autophagy genes → enhances mitophagy SIRT1/Atg5/Atg7: Promotes assembly of autophagy initiation complex AMPK: Phosphorylation at Thr172 → inhibits mTOR → maintains autophagic flux Nrf2–p62: Activates Nrf2 nuclear translocation → antioxidant defense → p62 feedback enhances autophagy	Neonatal rat cardiomyocytes hypoxia/reoxygenation model	↑LC3-II; ↑mitophagosomes; activated BNIP3L-mediated mitophagy; improved myocardial energy metabolism	[105–118]
Curcumin	Turmeric (Curcuma longa)	Nrf2/Keap1: Phenolic hydroxyl groups covalently bind Keap1 Cys151 → release and nuclear translocation of Nrf2 → activates antioxidant and autophagy-related genes SIRT1/AMPK/mTOR: Activates SIRT1 → deacetylates PGC-1*α* → activates AMPK → inhibits mTOR → enhances autophagy PI3K/Akt/mTOR: Inhibits excessive autophagy to prevent cardiomyocyte damage	Neonatal rat cardiomyocytes High-fat diet-induced diabetic cardiomyopathy model; DOX-induced cardiotoxicity model hypoxia/reoxygenation model	↑LC3-II/I; ↓p62; improved mitochondrial homeostasis; bidirectional regulation of autophagy; reduced cardiomyocyte apoptosis and left ventricular remodeling	[119–129]
Berberine	Coptis chinensis, Phellodendron amurense, Berberis vulgaris	AMPK/mTORC1: Activates AMPK → inhibits mTORC1 → enhances autophagic flux SIRT1/PGC-1*α*: SIRT1 deacetylates PGC-1*α* → restores mitochondrial function Parkin: Activates Parkin pathway → clears damaged mitochondria → improves energy metabolism	TAC pressure overload model; HFpEF mouse model	Reduced cardiac hypertrophy; restored mitochondrial homeostasis; improved myocardial function; decreased cardiomyocyte apoptosis	[130–144]
Palmatine	Coptis chinensis and other medicinal herbs	SIRT1/NF-κB/NLRP3/AMPK/mTOR/Nrf2/HO-1: Activates SIRT1 → induces protective autophagy; inhibits NF-κB/NLRP3 inflammation; activates AMPK/mTOR → promotes autophagy; activates Nrf2/HO-1 → enhances antioxidant defense	DOX-induced cardiomyopathy mouse model	Improved cardiac function; reduced inflammation and oxidative stress; promoted autophagy-mediated cardioprotection	[145–149]
Fucoxanthin	Brown algae (Undaria pinnatifida, Laminaria japonica)	AMPK/mTOR: Activates AMPK → inhibits mTOR → enhances autophagy SIRT1/PGC-1*α*: Promotes mitochondrial biogenesis and oxidative phosphorylation → enhances mitophagy Nrf2/HO-1: Reduces oxidative stress BNIP3/NIX: Induces mitophagy	Myocardial ischemia/reperfusion; high glucose-induced diabetic cardiomyopathy	↑LC3-II/Beclin-1; ↓p62; improved mitochondrial homeostasis and energy metabolism; reduced oxidative stress and myocardial injury	[150–161]
Astaxanthin	Algae (Haematococcus pluvialis), shrimp, crab	AMPK/mTOR: Activates AMPK → inhibits mTOR → initiates autophagic flux SIRT1/FOXO3a: Deacetylates FOXO3a → upregulates autophagy genes PINK1/Parkin: Promotes clearance of damaged mitochondria Nrf2/HO-1: Enhances antioxidant defense	DOX-induced cardiotoxicity; hypoxia/reoxygenation model	Enhanced autophagy and mitophagy; restored mitochondrial function; decreased ROS; improved myocardial injury	[162–167]

**Table 2:** Clinical transformation of natural compounds

Compound	HF model type	Major autophagy-related pathways	Animal efficacy summary	Human / Preclinical data	References
Quercetin	Diabetic cardiomyopathy rats, myocardial hypoxia models	AMPK/mTOR; SIRT1/TMBIM6; PI3K/Akt/FOXO3	Improved myocardial structure, ↑ LC3/Beclin1 expression, enhanced mitochondrial resistance to hypoxia	No clear human HF studies reported	[88–93]
Apigenin	Doxorubicin-induced myocardial injury, isoproterenol-induced models, ischemia–reperfusion (I/R)	PI3K/Akt/mTOR; SIRT1 pathway	Reduced Bax, increased Bcl-2, suppressed excessive autophagy, improved mitochondrial function	No specific human HF studies reported	[26, 94–105]
Resveratrol	HF animal models (including diabetic cardiomyopathy)	SIRT1/PGC-1*α*; AMPK	↑ ULK1/LC3 expression, enhanced mitochondrial autophagy, improved cardiac function	Limited preliminary human studies (mainly metabolic/cardiovascular adjunct)	[105–118]
Curcumin	Diabetic cardiomyopathy models	AMPK/JNK1; mTORC1 inhibition	Relieves Bcl-2/Bim inhibition of Beclin1, enhances autophagy, improves myocardial injury	Low bioavailability; minimal human HF studies	[119–129]
Berberine (BBR)	Aortic constriction; HFpEF; I/R models	PINK1/Parkin-mediated mitophagy	Upregulates PINK1/Parkin, improves mitochondrial autophagy flux and diastolic function	Some human metabolic/cardiac studies exist, but limited HF-specific data	[130–144]
Berberrubine	Cardiac aging models	PHB2-mediated mitophagy	Improves cardiomyocyte function in animal models; limited HF model data	No human HF data available	[145–149]
Fucoxanthin	Myocardial ischemia–reperfusion (MIRI) model	AMPK/GSK-3*β*/Nrf2; ferroptosis regulation	Significantly alleviates I/R injury, modulates autophagy and ferroptosis	No human HF studies reported	[150–161]
Astaxanthin	Pressure overload-induced cardiac hypertrophy	SIRT1/FOXO3	Moderately induces autophagy, improves mitochondrial function and cardiac hypertrophy	Small-scale human cardiovascular/metabolic studies exist, HF-specific data lacking	[162–167]

### Preclinical evidence and development prospects

Among polyphenols and flavonoids, resveratrol and quercetin are the most extensively investigated molecules with solid preclinical evidence. Resveratrol possesses high fat solubility and efficient transmembrane permeability due to its stilbene skeleton and 3,5-dihydroxy substitution pattern, facilitating its preferential activation of the SIRT1/PGC-1*α* axis and subsequent enhancement of mitochondrial quality control and autophagy-dependent energy metabolic improvement [168]. In contrast, quercetin relies on the strong electron-donor capacity conferred by its polyhydroxylated aromatic ring, making it more inclined to activate the AMPK/mTOR pathway and thereby enhance autophagy initiation and reduce oxidative stress [72]. Although apigenin is also a flavonoid, its distinct hydroxylation pattern and molecular configuration enable dual-pathway regulation; beyond activating AMPK/mTOR, it can also promote mitochondrial homeostasis and lysosomal substrate clearance through TFEB-mediated autophagy-lysosomal activation [104, 169], reflecting its multi-target and multi-level regulatory features. Based on current in vivo and in vitro evidence, resveratrol and quercetin may be regarded as lead compounds with comparatively robust preclinical support.

Among alkaloids, BBR can be readily localized to mitochondrial membranes because its quaternary ammonium structure confers strong cationic properties, allowing preferential accumulation in cardiomyocytes with high membrane potential. This property enables effective activation of AMPK/mTOR and SIRT1/PGC-1*α*-dependent mitophagy pathways [139, 143, 144]. Palmatine also exhibits multi-target regulatory potential along the SIRT1/AMPK/mTOR axis and demonstrates cardioprotective effects in doxorubicin-induced injury models [145, 149]. Although its protective effects are evident, the depth and breadth of its preclinical research remain more limited compared with polyphenols.

Marine-derived natural products such as fucoxanthin and astaxanthin possess strong membrane-embedding capability and free-radical-scavenging activity due to their polyene backbones and terminal hydroxyl groups. These structural characteristics contribute to the improvement of oxidative stress and autophagy flux [155, 163]. While current preclinical data are still preliminary, their unique structures and multi-target activities highlight considerable potential for further development, particularly when combined with nano-delivery systems or targeted delivery strategies.

## Challenges and future directions

The clinical translation of natural compounds for the intervention of HF still faces multiple obstacles. Although extensive basic research demonstrates that these compounds can modulate AMPK/mTOR, SIRT1/FOXO, and BNIP3/NIX pathways to improve myocardial energy metabolism and suppress apoptosis and inflammation, the transition from mechanistic studies to practical clinical application remains limited. For instance, although activation of the AMPK/mTOR axis improves cardiac function in animal models [170], the optimal intervention window in humans-considering individual energy status and drug exposure-remains uncertain. Likewise, the activity of the SIRT1/FOXO axis is tightly associated with aging and oxidative stress, and interindividual metabolic and genetic differences may lead to heterogeneous treatment responses [171]. Moreover, while BNIP3/NIX-mediated mitophagy exerts protective effects in ischemia–reperfusion injury, its sustained activation may induce cardiomyocyte death [37, 172].

Another key limitation arises from model selection. Current animal models predominantly simulate HFrEF, whereas the complex pathophysiological characteristics of HFpEF are insufficiently reproduced, resulting in discrepancies between preclinical efficacy and clinical outcomes in HFpEF patients [173]. Therefore, future research should adopt phenotype-stratified designs and integrate multi-omics approaches to elucidate compound–target–pathway networks, allowing more accurate definition of benefit windows and phenotype-specific responsiveness of autophagy regulation.

Pharmacokinetic constraints also significantly hinder clinical translation. Many natural compounds exhibit poor absorption, rapid metabolism, short plasma half-life, and difficulty maintaining stable therapeutic concentrations [174]. Curcumin, for example, shows cardioprotective potential in animal studies but suffers from extremely low oral bioavailability, markedly limiting clinical efficacy [175]. Marine-derived compounds pose additional challenges due to their structural complexity, high lipophilicity, and unique metabolic profiles. Strategies such as nano-delivery systems, liposomes, PLGA nanoparticles, and rational structural modification have therefore become essential approaches to enhance stability, targeting capability, and sustained drug release [13, 176].

At the clinical research level, although evidence remains limited, several natural compounds have already entered early trial stages. Curcumin has shown preliminary benefits in improving cardiac function, inhibiting inflammation, and modulating metabolism [177]. Compounds such as tanshinone and astragaloside have also demonstrated good safety profiles in phase II studies. However, most trials involve small sample sizes and short follow-up durations, and their endpoints mainly focus on metabolic or inflammatory indicators, lacking long-term clinical outcomes and mechanistic verification [178].

In the future, establishing multi-center, large-scale clinical research systems with dynamic biomarker monitoring will be crucial. Such systems will enable systematic evaluation of dose relationships, safety, and long-term efficacy, ultimately providing a more robust evidence base for the clinical application of natural compounds targeting autophagy.

## Conclusion

HF is a complex condition involving multiple cell types and signaling networks, characterized by disrupted myocardial energy metabolism, progressive fibrosis, and microcirculatory dysfunction. Autophagy plays a dual, context-dependent role: its appropriate activation facilitates the clearance of damaged mitochondria, preserves energy homeostasis, and limits apoptosis, whereas excessive activation or impaired autophagic flux may exacerbate mitochondrial loss and myocardial injury. Variations across disease stages, models, and cellular contexts contribute to inconsistent experimental conclusions, highlighting the pronounced spatiotemporal dependence of autophagy and the absence of a systematic definition of its protective and detrimental thresholds. This spatiotemporal specificity also suggests that autophagy is intricately regulated by upstream signaling networks rather than functioning as an isolated process. Crosstalk among the AMPK/mTOR, SIRT1/FOXO, and PINK1/Parkin pathways orchestrates energy balance, stress responses, and fibrotic remodeling. Their synergistic or antagonistic interactions may lead to divergent outcomes for the same intervention at different disease stages. To clarify the stage-specific role of autophagy during HF progression, future studies should adopt unified autophagy flux assessment criteria, cell type-specific intervention strategies, and dynamic analyses of dose–time relationships to dissect the integrated effects of signaling networks.

Natural compounds have emerged as promising multi-target autophagy modulators, yet their effects depend heavily on molecular structure, metabolic stability, and temporal patterns of action, resulting in occasional inconsistency across studies. For structurally complex molecules-particularly marine-derived compounds—the target specificity and structure–activity relationships remain insufficiently defined. Future research should integrate multi-omics, network pharmacology, and single-cell technologies to elucidate the systemic regulation of autophagy. Moreover, phenotype-stratified studies based on HFpEF and HFrEF, incorporating dynamic biomarker monitoring and long-term follow-up, will be essential for establishing a translational framework that bridges mechanistic discovery with precise therapeutic intervention.

The definition of abbreviations is shown in Appendix to Table 3.

**Table 3:** Appendix

Abbreviation	Full term
AICAR	5-Aminoimidazole-4-carboxamide ribonucleotide
AMPK	AMP-activated protein kinase
Atg5 / Atg7	Autophagy-related gene 5 / 7
ATG	Autophagy-related proteins
Bax	Bcl-2-associated X protein
Bcl-2	B-cell lymphoma 2
BNIP3	BCL2/Adenovirus E1B 19 kDa Protein-Interacting Protein 3
BNIP3L (NIX)	BCL2/Adenovirus E1B 19 kDa Protein-Interacting Protein 3-Like (NIX)
CFs	Cardiac fibroblasts
DOX	Doxorubicin
ECM	Extracellular matrix
eNOS	Endothelial nitric oxide synthase
FOXO / FoxO1 / FoxO3a / FOXO3	Forkhead box O transcription factors
FUNDC1	FUN14 domain-containing protein 1
GSK-3*β*	Glycogen synthase kinase-3 beta
HF	Heart failure
HFpEF	Heart failure with preserved ejection fraction
HO-1	Heme oxygenase-1
IL-11	Interleukin-11
I/R	Ischemia/Reperfusion
JNK1	c-Jun N-terminal kinase 1
Keap1	Kelch-like ECH-associated protein 1
LC3 (LC3-I / LC3-II)	Microtubule-associated protein 1A/1B-light chain 3
LPS	Lipopolysaccharide
MIRI	Myocardial ischemia–reperfusion injury
MMPs	Matrix metalloproteinases
mTOR / mTORC1	Mechanistic target of rapamycin / mTOR complex 1
NF-κB	Nuclear factor kappa-light-chain-enhancer of activated B cells
NLRP3	NOD-like receptor pyrin domain-containing protein 3
NO	Nitric oxide
Nrf2	Nuclear factor erythroid 2-related factor 2
p70S6K	Ribosomal protein S6 kinase beta-1
PHB2	Prohibitin 2
PI3K/Akt	Phosphatidylinositol 3-kinase / Ak strain transforming
PGC-1*α*	Peroxisome proliferator-activated receptor gamma coactivator 1-alpha
PINK1	PTEN-induced kinase 1
Raptor	Regulatory-associated protein of mTOR
ROS	Reactive oxygen species
SIRT1	Sirtuin 1
TAC	Transverse aortic constriction
TGF-*β*1	Transforming growth factor-*β*1
TIMPs	Tissue inhibitors of metalloproteinases
TFEB	Transcription factor EB
TMBIM6	Transmembrane Bax inhibitor motif-containing protein 6
ULK1	Unc-51-like autophagy-activating kinase 1
Wnt	Wingless-related integration site signaling
*α*-SMA	Alpha-smooth muscle actin

## Data Availability

No datasets were generated or analysed during the current study.
